# A co-designed program for better sleep in Australian First Nations adolescents: protocol for the Let’s Yarn About Sleep adolescent sleep health program

**DOI:** 10.1093/sleepadvances/zpaf012

**Published:** 2025-02-22

**Authors:** Yaqoot Fatima, Roslyn Von Senden, Romola S Bucks, Caitie Ashby, Daniel P Sullivan, Simon S Smith, Sarah Blunden, Stephanie Yiallourou, Peter R Eastwood, Abdullah A Mamun, Lisa McDaid, Jen Walsh, Mina Kinghorn, Azhar H Potia, Sharon Varela, Stephanie King, Shaun Solomon, Markesh Fanti, Timothy C Skinner

**Affiliations:** Thompson Institute, University of the Sunshine Coast, Sunshine Coast, Queensland, Australia; ARC Centre of Excellence for Children and Families over the Life Course, Brisbane, Queensland, Australia; Thompson Institute, University of the Sunshine Coast, Sunshine Coast, Queensland, Australia; School of Psychological Science, University of Western Australia, Perth, Western Australia, Australia; School of Population and Global Health, University of Western Australia, Perth, Western Australia, Australia; Thompson Institute, University of the Sunshine Coast, Sunshine Coast, Queensland, Australia; Thompson Institute, University of the Sunshine Coast, Sunshine Coast, Queensland, Australia; ARC Centre of Excellence for Children and Families over the Life Course, Brisbane, Queensland, Australia; Department of Psychology, Prince Charles Hospital, Brisbane, Queensland, Australia; ARC Centre of Excellence for Children and Families over the Life Course, Brisbane, Queensland, Australia; ARC Centre for Excellence for the Digital Child, Brisbane, Queensland, Australia; Child Health Research Centre, The University of Queensland, Brisbane, Australia; Appleton Institute of Behavioural Science, Central Queensland University, Adelaide, South Australia, Australia; Turner Institute for Brain and Mental Health, School of Psychological Sciences, Monash University, Clayton, Victoria, Australia; Health Futures Institute, Murdoch University, Perth, Western Australia, Australia; ARC Centre of Excellence for Children and Families over the Life Course, Brisbane, Queensland, Australia; Poche Centre for Indigenous Health, The University of Queensland, Brisbane, Australia; Institute for Social Science Research, University of Queensland, Brisbane, Queensland, Australia; Department of Pulmonary Physiology and Sleep Medicine, Sir Charles Gairdner Hospital, Perth, Western Australia, Australia; Centre for Sleep Science, University of Western Australia, Perth, Western Australia, Australia; Thompson Institute, University of the Sunshine Coast, Sunshine Coast, Queensland, Australia; ARC Centre of Excellence for Children and Families over the Life Course, Brisbane, Queensland, Australia; Queensland Brain Institute, University of Queensland, Brisbane, Queensland, Australia; Murtupuni Centre for Rural and Remote Health, James Cook University, Mount Isa, Queensland, Australia; Murtupuni Centre for Rural and Remote Health, James Cook University, Mount Isa, Queensland, Australia; Murtupuni Centre for Rural and Remote Health, James Cook University, Mount Isa, Queensland, Australia; Thompson Institute, University of the Sunshine Coast, Sunshine Coast, Queensland, Australia; Australian Centre for Behavioural Research in Diabetes, Deakin University, Melbourne, Victoria, Australia; Institute of Psychology, University of Copenhagen, Copenhagen, Denmark

**Keywords:** sleep health, First Nations, adolescents, social–emotional well-being

## Abstract

The first-ever comprehensive report on the sleep health of Aboriginal and Torres Strait Islander peoples (hereafter referred to as First Nations Australians) highlighted an 18% prevalence of poor sleep in First Nations youth. While sleep health is important across the lifespan, adolescence is a critical life stage with increased vulnerability to poor sleep. In adolescents, pubertal changes, social and academic commitments, and peer pressure significantly increase the risk of poor sleep, which often results in social and emotional well-being (SEWB) issues. In First Nations adolescents, high rates of SEWB issues demand effective prevention and management strategies. Evidence from non-First Nations adolescents suggests that timely prevention, identification, diagnosis, and management of poor sleep help reduce the risk and severity of SEWB issues in First Nations adolescents. A research program is proposed to be called “Let’s Yarn About Sleep,” which will co-design, deliver, and evaluate a tailored sleep improvement program for Australian First Nations adolescents (12–18 years). Co-design workshops will be conducted with First Nations community Elders, parents and carers, youth, and First Nations service providers to develop the sleep health program. The program will also include training Aboriginal Youth Workers (AYWs) to deliver the sleep health program. The program evaluation will be based on a mixed methods design, using self-reported (survey tools and focus group discussions) and technology-based measures (actigraphy data) to measure changes in First Nations adolescents’ sleep and SEWB. The evaluation will focus on the impact of training AYWs on program delivery and uptake.

**Statement of Significance**

The Let’s Yarn About Sleep study is the first sleep health program based on strengths-based, culturally informed approaches to promoting sleep health in First Nations communities. Community governance, privileging First Nations’ worldviews and culturally responsive integration of mainstream sleep science knowledge and First Nations cultural knowledge on sleep health, will positively impact program acceptance and effectiveness. Investing in community capacity-building, i.e. training Aboriginal Youth Workers, will contribute to sustainable sleep health promotion in First Nations communities. Program feasibility, acceptability, and effectiveness evaluation will be based on a clear evaluation framework with indicators identified by First Nations communities and stakeholders.

## Introduction

Aboriginal and Torres Strait Islander peoples, custodians of the longest-surviving culture in the world, are the Indigenous peoples of Australia, comprising two similar but distinct cultural groups, Aboriginal Australians and Torres Strait Islander peoples [1]. These groups encompass over 250 language groups, including hundreds of dialects and unique expressions of cultural identity and practice. In total, they represent approximately 3% of the Australian population [2, 3]. Although cultural practices and expressions are unique and heterogeneous, core concepts of family, kinship relatedness, and relationality, connections to Country and culture underpin Aboriginal and Torres Strait Islander peoples’ worldviews and philosophies of cultural identity, practice, and social organization [4]. While acknowledging the nuances and diversity of Aboriginal and Torres Strait Islander cultures and identities across Australia, we will use First Nations Australians as a collective term for Aboriginal and Torres Strait Islander peoples hereafter.

The European settlement of Australia led to the loss of sovereignty and dispossession of lands, waterways, customary law, and cultural heritages for First Nations Australians [5]. The historical and continuing impacts of colonization, alongside intergenerational trauma, racism, and systemic disadvantage, profoundly affect the education, housing, employment, health and social–emotional well-being (SEWB) outcomes of First Nations peoples [6]. In Australian First Nations communities, SEWB represents a multidimensional concept of health encompassing mental health and other key domains of health and well-being such as connection to land or “country,” culture, spirituality, ancestry, family, and community [7]. Post-colonization, loss of cultural identity, and connection to land have further exacerbated SEWB issues, as cultural continuity is a crucial protective factor for SEWB [8]. Despite this, Australian First Nations cultures remain strong, vibrant, and resilient and continue to be expressed through language, cultural practices, and identity.

In recent years, First Nations health research has witnessed a much-needed positive shift from a “problem-centric” approach to a “strengths-based solution-centric” approach privileging First Nations’ worldviews to identify opportunities for early intervention [9]. This shift also aligns with the imperative to address health inequities related to sleep health. Disproportionate rates of poor sleep health in First Nations communities, primarily driven by systemic and social determinants such as intergenerational trauma, housing issues, and racism, highlight the pressing need to achieve sleep health equity—ensuring all individuals have access to opportunities and support for optimal and restorative sleep experiences essential for health and well-being. For First Nations peoples, this also entails co-designing culturally safe, community-led sleep health interventions responsive to their cultural contexts, values, and needs.

While the risk of poor sleep is increasing across all age groups, adolescents are particularly vulnerable due to pubertal changes, increased academic, social, and extracurricular demands, and constant access to electronic devices [10]. Poor sleep in adolescents is strongly linked with their mood, behavior, school performance, and SEWB [11]. A third of First Nations young people aged 15–24 experience very high levels of psychological distress, and suicide and anxiety disorders are the leading disease burden in this cohort [12]. Considering the high rates of SEWB issues in First Nations adolescents and the protective role of sleep against these issues, achieving sleep health equity has become a priority. To minimize the downstream impact of poor sleep on health, well-being, and other life outcomes, community members and healthcare providers are advocating for investing in sleep health for First Nations adolescents as a priority [12, 13].

The use of behavioral sleep programs as a transdiagnostic approach for a range of sleep and mental health problems has been found to be an effective treatment approach [14]. However, while mainstream sleep health interventions can improve sleep in non-First Nations contexts, they may be less effective in First Nations communities, as unique cultural and societal contexts shape sleep health perceptions [15]. A nuanced understanding of sleep health is particularly important for First Nations peoples, for whom health is intertwined with their connection to country, culture, spirituality, and ancestry [16]. Therefore, western sleep health interventions, which are not centered on the strengths of First Nations knowledges and culture and do not acknowledge relevant contextual challenges and social determinants of health, are inherently ineffective in First Nations communities. Furthermore, limited local capacity-building and community advocacy can negatively impact the acceptability and uptake of health programs, which may lack cultural safety if not community-led. As such, in addition to the availability of culturally appropriate programs, sleep health promotion in First Nations communities also requires strengthening the local community’s capacity and developing local champions for culturally appropriate delivery of sleep health programs.

Consultations with community members in Mount Isa, a remote town in Northwest Queensland (Australia), helped the research team “co-define” sleep health from their First Nations perspectives and “co-identify” the causal supportive factors linked with poor sleep in First Nations communities [17]. These consultations identified that dreaming and harmony within mind, body, and spirit play a crucial role in First Nations peoples’ conceptualizations of sleep health. Participants also reported that lack of awareness of sleep health, neighborhood noise and crimes, lack of safe and comfortable sleeping spaces, and problem trivialization by primary care services are some of the main contributors to poor sleep in First Nations communities [17]. This pilot work identified a high unmet need to address the sleep health of First Nations peoples and established the groundwork for the proposed program. Community Elders proposed the program name—Let’s Yarn About Sleep. The term “Yarn” holds great significance in Australian First Nations culture. It is a conversational process that involves storytelling in a culturally safe environment, offering opportunities for reflection on one’s actions and those of others [18].

The Let’s Yarn About Sleep program draws upon the community “co-defining” and “co-identifying” stages to “co-design” a sleep health program that will be respectful and responsive to cultural norms and contextual factors affecting sleep health. The last stage of the program lifecycle will be to “co-develop” the evaluation framework, where we will seek community input to develop the measures for assessing the program’s success and effectiveness from the perspective of First Nations peoples.

### Project aim and objectives

The overarching goal of the Let’s Yarn About Sleep project is to co-design, deliver, and co-evaluate a sleep health program for First Nations adolescents in remote Australia. The specific objectives of this project are to:

1. Co-create a sleep health program for First Nations adolescents
2. Train Aboriginal Youth Workers (AYWs) as “Sleep Coaches” to deliver the program.
3. 3, Evaluate the program’s acceptability, feasibility, and effectiveness in improving First Nations adolescents’ sleep health and SEWB.

## Methods

### Design and setting

This Let’s Yarn About Sleep program will be designed and delivered in Mount Isa (Australia). Approximately a quarter of the population (24%) in Mount Isa identify as First Nations Australians (compared to 4.6% across the state of Queensland and 3.3% across Australia) [19] and 12.4% of the Mount Isa population is aged 12–19 years [20].

### Program framework

The proposed program is based on the Nukal Murra (Plenty Hands) SEWB framework [21], a strengths-based approach tailored to improve health outcomes for rural and remote First Nations communities. The framework is based on the Gayaa Dhuwi (Proud Spirit) declaration. It recognizes the resilience of First Nations peoples, including connection to country and culture, spirituality, ancestral ties, resilience, kinship, community leadership, and governance [22].

### Strengths-based solution-centric approach

Deficit narratives and problem-centric approaches to solving health issues may not always address the systemic and social determinants of health and, therefore, present a risk of perpetuating stigma or disregarding community strengths [23]. Understandably, these approaches often fail to engage end users in the research process and deliver solutions that do not meet the community’s needs and expectations. Strength-based approaches, particularly in the context of First Nations health research in Australia, are rooted in First Nations communities’ strengths, resilience, and cultural knowledges, rather than deficits or challenges [24]. This approach embeds community leadership and deep partnerships that ensure research objectives align with community priorities, are guided by local knowledge, and leverage community assets. Integrating an action-ontological approach covering the community’s social, emotional, and spiritual wellness is a key focus of a strengths-based research approach, which strongly aligns with the holistic nature of First Nations Australians’ ways of knowing, being, and doing [7]. Using a solution-centric strengths-based approach facilitates focus on the strengths of the community, challenges stereotypes, helps decolonize health research, and is more likely to produce research outcomes that are culturally responsive and meet the needs of end users [25]. Guided by the success of and community response to strengths-based approaches, the Let’s Yarn About Sleep program will also include this approach in the program design, delivery, and dissemination.

### Project governance

This project adopts a multilayered governance approach to foster effective collaboration, accountability, decision-making, self-determination, and ownership. A Community Elders steering group will assist the project team in actively engaging with the community, conducting community consultations, recruiting participants, exchanging information, and using community feedback to improve the program. An advisory group of service providers, involving staff from local schools, health, law enforcement, youth welfare, and housing services, will be established for engaging service providers and evidence sharing. The research team will also be guided and supported by First Nations researchers, providing essential First Nations academic mentorship and leadership.

### Participants

For co-design workshops, First Nations Australian young people aged ≥12–18 years, and their parents or carers/guardian, or stakeholders associated with services involved with housing, education, employment, law and safety, health and well-being of First Nations youth, will be invited to join a workshop.

For sleep health program participation, First Nations adolescents aged >12–18 years experiencing poor sleep and/or mental health will be invited to participate. Poor sleep health will be operationalized by self-identifying the presence of at least one of the following issues: <8 hours sleep/night (the lower bound of appropriate sleep duration for teenagers by consensus of the Sleep Health Foundation [26]), difficulty in sleep initiation or maintenance, waking up too early, daytime fatigue and sleepiness over the preceding 4-week period. Mental health inclusion criteria will be determined through depression/anxiety items of the Strong Souls questionnaire [19]. Informed consent from self and parents/carers will also be required. Participants identified as having high suicide risk (indicated by the Strong Souls suicide items), clinically diagnosed severe and morbid obesity, neuromuscular disease, cognitive impairment, or lung diseases will be excluded from the study due to the risk of poor adherence and the need for specialist medical or psychiatric care [27].

#### Recruitment and consent.

The program will be promoted on media and social media channels, in primary care and youth social and emotional well-being services, and in schools to target potential participants. The AYWs will facilitate contact and engagement with First Nations adolescents, parents and community elders and liaise with youth services to recruit and retain adequate participants for co-design workshops, program delivery and evaluation activities. The project promotion activities will invite community members and service providers to participate in co-design workshops, specifically targeting First Nations adolescents to join the sleep health program. Written informed consent will be obtained from all participants and their parents/guardians. Cultural protocols will be followed at all stages, and the Elders steering group will guide the recruitment process.

#### Participant incentives.

A high value will be placed on maintaining the sample through relationship building and compensation of participants. Participants will receive a $50 (AUD) gift voucher at each pre–post assessment, and midway through the program (completion of session three) in recognition of the participant’s time commitments to attend the program in addition to the assessments. The AYWs will leverage their relationships with First Nations adolescents and parents to build and maintain community connections and engage participants and families to ensure participant engagement and retention.

### Let’s Yarn About Sleep program activities

The program comprises the following three activities:

1. **Training of AYWs:** AYWs are mentors, role models, and advocates for the young people within First Nations Communities. They bring a deep understanding of the community and its cultural and contextual needs to understand issues regarding the First Nations’ young people. By training AYWs as “Sleep Coaches,” we aim to leverage their cultural knowledge, community ties, and ability to work with young people to maximize the program reach, engagement and uptake. AYWs in the community will be recruited as project officers and receive on-the-job training for delivering the sleep health program. In addition to receiving scientific and clinical training on sleep health promotion, the AYWs will also receive cultural training for effective sleep health promotion in the community. The Elders steering group members will share traditional and cultural knowledge on healthy sleep that complements mainstream sleep health knowledge focusing on sleep needs, the importance of healthy sleep, the link between sleep and SEWB, sleep disorders, measuring sleep, strategies for sleep optimization, and management of behavioral sleep problems in adolescents. The community co-design workshops will offer opportunities to discuss sleep health training and identify community views on additional elements to strengthen the training of the AYW’s to ensure culturally responsive sleep health promotion. In the project’s second year, follow-up training on program facilitation and evaluation will be offered to support the AYWs further.
2. **Co-design of sleep health improvement program and co-development of program evaluation measures:** The co-design process will be based on community-based participatory research and knowledge co-creation [28]. First Nations community members and service providers will be invited to participate in co-design sessions (up to 60 minutes each), where creative and expressive methods (such as storytelling, painting, and weaving) will be used to encourage the participants to share their ideas for the program. Embedding these methods in workshops will enable meaningful participation and embedding First Nations peoples’ rich cultural traditions in the data collection process. Participants will be asked to (1) share their views on the elements of an effective and sustainable sleep health program for First Nations adolescents, mainly focusing on culturally appropriate sleep health promotion, strategies to provide safe sleeping spaces, opportunities to offer catch-up sleep, service access, referral pathways, and support for adolescents with poor sleep; (2) identify whether there are groups of First Nations adolescents requiring specialized care and support with improving their sleep health; (3) share their ideas for program content, structure, and delivery strategy; (4) consider and discuss the potential relevance and effectiveness of strategies shared by the research team (based on findings from our research and mainstream sleep health knowledge on sleep health [29–38], clinical knowledge and expertise, and elements of successful sleep health programs in non-First Nations populations) in improving the sleep health of First Nations adolescents; (5) discuss their expectations from the program; and (6) create their ideal sleep health program and share the story of why they prefer it. Based on all community consultation data and the high level of input from the steering and advisory groups, the research team will develop a preliminary framework of the program elements, features, contents, and delivery strategy. Community members will be asked to provide feedback following an iterative feedback process. This process will be repeated (over 3 months) until a broad agreement is reached. The participants will also be asked to identify and define the constructs for assessing program success and effectiveness regarding sleep health. To initiate the conversation on sleep health, we will use contemporary definitions of key sleep health concepts such as sleep health knowledge (operationalized as knowledge and understanding of optimal sleep experience and its impact), sleep hygiene (operationalized as behavioral and environmental factors impacting sleep experience), and sleep habits (operationalized as sleep patterns, including bedtime, wake time, and variability in sleep timing). However, to ensure these definitions are culturally responsive, they will be presented in community co-design workshops for discussion and adaptation. This process will allow us to refine the operationalization of these terms from First Nations perspectives.
3. We will also share standard validated tools for assessing sleep-related practices, beliefs, and attitudes (Sleep Knowledge Questionnaire [39]), sleep-facilitating and sleep-inhibiting practices (Adolescent Sleep Hygiene Scale [40] assessing), and poor sleep (Insomnia Severity Index [41], Pittsburgh Sleep Quality Index [42]) with the participants to enrich discussion on program measure development. A key focus of co-design workshops will be to adapt/develop culturally appropriate sleep health questionnaires, enabling the robust and culturally responsive collection and reporting of sleep health data. This approach is critical to accommodate First Nations communities’ diversity and ensure that the tools are meaningful and acceptable. Based on participant-defined constructs and criteria for program effectiveness, sleep health and hygiene (healthy sleep habits) assessment tools will be developed for community approval. It is not anticipated that the co-designed study materials and measures will need to be translated from English into First Nations traditional languages. In the 2016 Australian Census, 93% of First Nations Australians living in Mount Isa and surrounding communities reported speaking English at home [43]. Finally, the steering and advisory group members will be asked to help assess the measures’ cultural appropriateness, face, and content validity.
4. **Program delivery:** The AYWs will use the program and resources developed through the community co-design to run the sleep health sessions for First Nations adolescents. Based on our knowledge and available evidence [44], we anticipate that six sessions of about 45–60 minutes will be needed for effective knowledge transfer.

### Study outcomes and analytic strategies

The Ngaa-bi-nya framework for First Nations health and social program evaluation and co-designed evaluation tools will be used in evaluation [45].

#### Process outcomes.

The change in AYWs capacity will be assessed by reviewing their reflections on participating in the program and self-perceived improvement in their knowledge of sleep health and competency in youth sleep health promotion. The program participants will also be asked to provide feedback on the performance of AYWs and the impact of their training at the community level.

Context-specific barriers and facilitators affecting program delivery and improvement opportunities will be assessed by conducting systematic and regular document analysis, including a review of the documents related to program participation and implementation. Interviews will be conducted with key stakeholders to identify barriers and enablers affecting program delivery, perceptions of the impact on AYW capacity-building, achievements of the program against the original objectives, strengths and limitations, and opportunities for improvement.

#### Program outcomes.

Program outcomes will include changes in sleep health knowledge, self-reported sleep hygiene, sleep habits, and SEWB measured at baseline and post-program points.

##### Self-reported outcomes

Participants will complete a daily electronic or paper-based sleep diary. It will capture self-reported times of sleep onset and offset, total sleep duration, wake after sleep onset, and perceived overall sleep quality. Sleep health assessment tools will be co-designed with community members to ensure a culturally responsive and strengths-based assessment of participants’ sleep health and habits.

Sociodemographic data (age, biological sex, education, family income), environmental factors (crowding and neighborhood safety), health, social support, and lifestyle (smoking, alcohol consumption, any substance abuse or misuse, prescription and non-prescription drugs, diet, physical activity, TV, and electronic communication devices use) will also be collected from participants at baseline. Data will also be collected again upon program completion (week 10) to assess changes in sleep hygiene, sleep health, and lifestyle. To assess participants’ sleep knowledge, they will complete a brief knowledge assessment related to sleep hygiene and adolescents’ general sleep health needs.

Psychological distress and resilience will be assessed using the shortened version of the Strong Souls questionnaire [46], a validated screening tool for SEWB in First Nations peoples. Strong Souls has been demonstrated to have construct validity for SEWB similar to that of the Westerman Aboriginal Symptoms Checklist for Youth (WASC-Y), and the face and cultural validity items on Strong Souls were confirmed by First Nations young people. Development of Strong Souls found the measures loads onto four factors (anxiety, depression, suicide risk, and resilience), and with good internal reliability across all factor and item levels (Cronbach’s α ≥ 0.7) [46].

##### Technology-based Measures

To assess sleep, each participant will wear an actigraphy device (GENEActiv Original; Activinsights Ltd, Cambridgeshire, UK) on the nondominant wrist for seven nights before and after completion of the program. The GENEactiv device is a micro-electro-mechanical accelerometer, which records movement, wrist angle, wrist temperature, and light. This will be used to derive sleep parameters including: sleep efficiency, pattern/timing (e.g. bed and wake times, week/weekend variability), and sleep duration. Using validated open-source scoring software (GGIR) [47], with manual marking of sleep windows and off-wrist periods as determined by a Research Assistant who is appropriately trained in actigraphy analysis, with data and output reviewed by a board certified Behavioral Sleep Medicine specialist and Clinical Psychologist (author D.P.S.). Actigraphy measures will be enhanced and confirmed by electronic, or paper sleep diaries completed by participants. Wrist-worn actigraphy is a validated and commonly used method to assess sleep–wake patterning in adolescents [48, 49] and has been previously used with high acceptability by First Nations youth in remote communities [38].

### Sample size

The lack of prior research compromises formal sample size and power calculation. However, based on sleep health program effect sizes in non-First Nations adolescent populations, a total sample of 96 First Nations adolescents is required to detect a clinically meaningful effect size change in primary (0.7 units in sleep health awareness, 0.6 units in sleep hygiene, 0.5 unit in sleep quality), and secondary outcomes (0.3 unit in SEWB) when power and alpha levels are set at 0.8 and 0.05, respectively. Accounting for 10% attrition, a minimum of 113 participants will be needed for the study [50, 51]. This sample size is similar to that of quantitative and qualitative studies in sleep of American First Nations adolescents by Troxel and Dong’s group [52, 53].

### Statistical analysis

Statistical methods, including mixed models, will be utilized for assessing post-program changes [47, 54]. Mixed models are particularly well-suited for this analysis as they allow for the inclusion of both fixed effects, such as time and intervention, and random effects, such as individual or group variability, which is essential given the structure of the repeated measures. This approach is robust to missing or unbalanced data, a common issue in community-based research. Sleep parameters derived from actigraphy will be analyzed using paired sample *t*-tests for baseline and follow-up, including total sleep time, sleep efficiency, wake after sleep onset, average sleep onset and wake times, and sleep regularity index. Inductive thematic analysis for qualitative data will be used to elicit meaning, gain understanding, and develop empirical knowledge [55]. The expertise of First Nations researchers on our team and First Nations cultural mentors will provide a crucial perspective in interpreting and analyzing the findings.

### Ethics and dissemination

This project has been reviewed and approved by the University of Queensland’s Human Research Ethics Committees (2020/HE002899). The research findings will be broadly communicated across health services, community organizations, youth services, and state policymakers. They will also be presented at community symposiums, conferences, and peer-reviewed publications. Additionally, stakeholder workshops and community sessions will be conducted, and media and internet publications will be used to maximize the reach and uptake of this work outside academia.

## Discussion

Globally, the sleep health of First Nations peoples is significantly under-researched [37], with an even greater lack of understanding of the sleep health of First Nations peoples in Australia [56]. It is, therefore, unsurprising that preventive and management strategies for SEWB issues in First Nations communities have not yet utilized the potential benefits of implementing culturally responsive sleep health programs. Addressing the gaps in the current evidence base and sleep health promotion initiatives, the Let’s Yarn About Sleep program is unique in many ways. For example, drawing on the knowledge and success of “co-defining” and “co-identifying” processes, we are engaging the community to “co-design” a sleep health improvement program that is guided by First Nations worldviews and knowledge that will offer valuable insights to guide community engagement approaches used by researchers and agencies more broadly. The program will offer co-designed sleep health screening tools and sleep health resources to promote sleep health in First Nations adolescents. These tools and resources will integrate knowledge on social determinants of health, First Nations cultural knowledge and conceptualization of sleep health, and clinical knowledge on sleep science. Since such resources are lacking in the current provision of sleep health services, these tools will help clinicians discuss sleep health issues in a culturally safe manner and use resources that align with the First Nations’ worldview and are responsive to their lived experience to offer culturally responsive sleep health.

Considering the absence of such a program and sleep health resources for First Nations adolescents, who experience a disproportionately high rate of SEWB issues [12], the program outputs will likely have immense benefits for First Nations SEWB services and the sleep research community.

Further, despite the clear links between sleep health and mental health, reliable literature exploring sleep health outcomes for First Nations Australians is lacking [57, 58]. Western medicine approaches generally fail to consider the cultural strengths and contextual challenges First Nations Australians face in achieving healthy sleep [16, 58]. Incorporating First Nations knowledges and perspectives is essential to providing culturally responsive and effective sleep health support. Development and evaluations of existing sleep health evaluation tools, such as the Top End Sleepiness Scale, have reiterated the importance of creating and adopting culturally safe health and research tools for First Nations Australians [59]. Similarly, First Nations health workforce training and capacity-building are integral to providing culturally safe healthcare in Australia [60]. While sleep health research for First Nations peoples in Australia is a burgeoning area, training to develop a First Nations sleep health workforce will be crucial for effective sleep health research, service delivery, and achieving sleep health equity in First Nations communities.

### Limitations and future directions

The exclusion of participants at high risk of suicide and/or with clinically diagnosed severe morbid obesity, neuromuscular disease, cognitive impairment, or lung diseases means that the results of the study will not be generalizable to these populations. Due to the lack of a control group, it will be challenging to attribute improvements in sleep and SEWB solely to the program. Furthermore, this project is focused on one community only, thus limiting the appropriateness of its findings to broader Australian contexts and other First Nations communities. However, while the First Nations Australian cultures are highly diverse, the project’s findings will likely provide a useful foundation for building sleep knowledge and a roadmap for creating future culturally responsive programs in other First Nations communities.

In conjunction with leadership from the Mount Isa community and partnerships with key organizations, this project is already driven by continuous community engagement to lead, co-define, and co-design project activities and maximize the program’s value and translational impact. Community engagement and training of health professionals are central to this project and are intended to facilitate long-term improvements to the quality and provision of culturally responsive care in Australia.

## Data Availability

Data will be available on request, subject to approval from the study’s Data Governance Group to ensure data reuse is aligned with Indigenous data sovereignty principles. Data requests will also be subject to the requesting researcher having obtained separate approvals from their institutional Human Research Ethics Committee.

## Patient and Public Involvement

This pilot project is grounded in co-design and strengths-based methodologies and requires significant community engagement and involvement throughout all project stages.

## Registration Details

This study was not preregistered.
